# Incidence and prevalence of asthma, COPD, and ILD across England, Wales, and South-East Scotland – a harmonised approach to data analysis

**DOI:** 10.23889/ijpds.v9i5.2723

**Published:** 2024-09-18

**Authors:** Hywel Evans, Sean Scully, Sara Hatam, Sarah Cook, Hannah Whittaker, Costantinos Kallis, Ian Farr, Chris Orton, Aziz Sheikh, Jennifer Quint

**Affiliations:** 1 SAIL Databank, Population Data Science, Swansea University Medical School, UK; 2 Usher Institute, The University of Edinburgh; 3 Primary Care and Public Health, School of Public Health, Imperial College London, UK

## Objective

This study aimed to create harmonised datasets for asthma, chronic obstructive pulmonary disease (COPD), and Interstitial Lung Disease (ILD), and subsequently use them to calculate the incidence and prevalence of these diseases across three UK nations (England, Wales, and South-East Scotland).

## Approach

Harmonised research-ready datasets were created using three electronic healthcare record databases. Records were selected between 01/01/2004 and 31/12/2019, with yearly age and sex standardised incidence and prevalence rates calculated.

## Results

In 2019, the incidence rates for asthma were: England, 0.89 (95%CI: 0.88–0.90); Wales, 0.66 (0.65–0.68); and South-East Scotland, 0.67 (0.64–0.71), which were all lower than in 2004. Incidence rates of COPD in England were 0.83 (0.82–0.85) and 0.67 (0.65–0.69) in Wales, which were both lower than in 2004, but Scotland remained similar at 1.06 (0.99–1.13). Incidence rates of ILD were: England, 3.27 (3.05–3.50); Wales, 1.39 (1.27–1.53); and Scotland, 1.63 (1.36–1.95), which were all higher than in 2004. Between 2004 and 2019, the prevalence of all three diseases increased across all three nations.

## Conclusions

Solving differences in coding between CPRD, SAIL Databank, and DataLoch databases enabled comparable cohorts to be created and the first harmonised UK estimates of incidence and prevalence for asthma, ILD, and COPD.

## Implications

Harmonised studies of asthma, COPD, and ILD across the UK can now be performed. The code produced is publicly available to facilitate future research, which can be adapted for other conditions and databases.

